# Challenges in the Definitive Diagnosis of Niemann–Pick Type C—Leaky Variants and Alternative Transcripts

**DOI:** 10.3390/genes14111990

**Published:** 2023-10-25

**Authors:** Marisa Encarnação, Isaura Ribeiro, Hugo David, Maria Francisca Coutinho, Dulce Quelhas, Sandra Alves

**Affiliations:** 1 Research and Development Unit, Department of Human Genetics, National Institute of Health Doutor Ricardo Jorge, INSA I.P., Rua Alexandre Herculano, 321, 4000-055 Porto, Portugal; marisa.encarnacao@insa.min-saude.pt (M.E.); hugo.david@insa.min-saude.pt (H.D.); francisca.coutinho@insa.min-saude.pt (M.F.C.); 2 Center for the Study of Animal Science-Instituto de Ciências, Tecnologias e Agroambiente da Universidade do Porto, CECA-ICETA, University of Porto, Praça Gomes Teixeira, Apartado 55142, 4051-401 Porto, Portugal; 3 Associate Laboratory for Animal and Veterinary Sciences, AL4AnimalS, Faculdade de Medicina Veterinária Avenida da Universidade Técnica, 1300-477 Lisboa, Portugal; 4 Laboratório de Bioquímica Genética, Serviço de Genética Laboratorial, Centro de Genética Médica Jacinto Magalhães, Centro Hospitalar e Universitário de Santo António (CHUdSA), 4099-001 Porto, Portugal; isauraribeiro.cgm@chporto.min-saude.pt (I.R.); dulce.quelhas@chporto.min-saude.pt (D.Q.); 5 UMIB-Unit for Multidisciplinary Research in Biomedicine, ICBAS, University of Porto, 4099-002 Porto, Portugal; 6 ITR—Laboratory for Integrative and Translational Research in Population Health, 4050-600 Porto, Portugal; 7 Biology Department, Faculty of Sciences, University of Porto, Rua do Campo Alegre, 4169-007 Porto, Portugal

**Keywords:** Niemann–Pick type C, splicing variants, leaky variants, *NPC1* gene, molecular diagnosis

## Abstract

Niemann–Pick type C (NPC, ORPHA: 646) is a neuro-visceral, psychiatric disease caused predominantly by pathogenic variants in the *NPC1* gene or seldom in *NPC2*. The rarity of the disease, and its wide range of clinical phenotypes and ages of onset, turn the diagnosis into a significant challenge. Other than the detailed clinical history, the typical diagnostic work-up for NPC includes the quantification of pathognomonic metabolites. However, the molecular basis diagnosis is still of utmost importance to fully characterize the disorder. Here, the authors provide an overview of splicing variants in the *NPC1* and *NPC2* genes and propose a new workflow for NPC diagnosis. Splicing variants cover a significant part of the disease-causing variants in NPC. The authors used cDNA analysis to study the impact of such variants, including the collection of data to classify them as leaky or non-leaky pathogenic variants. However, the presence of naturally occurring spliced transcripts can misdiagnose or mask a pathogenic variant and make the analysis even more difficult. Analysis of the *NPC1* cDNA in NPC patients in parallel with controls is vital to assess and detect alternatively spliced forms. Moreover, nonsense-mediated mRNA decay (NMD) analysis plays an essential role in evaluating the naturally occurring transcripts during cDNA analysis and distinguishing them from other pathogenic variants’ associated transcripts.

## 1. Introduction

Lysosomal storage disorders (LSDs) are a group of about 70 inherited diseases, most of which are quite rare and present with vast clinical heterogeneity, ranging from severe, early-onset diseases to milder forms, of later onset. This remarkable variability may be observed not only between different diseases from the same group but also—and most importantly—amongst patients suffering from the same exact disease. Overall, this clinical heterogeneity has a direct impact on their diagnosis. Over the past several years, the number of available treatments for patients with LSDs has rapidly increased, namely, enzyme replacement and substrate reduction therapies, the use of molecular chaperones, gene therapy, and bone marrow transplant, among others [1]. Nevertheless, molecular diagnosis is the ultimate and essential step to provide access to therapy. The identification of biallelic nonsense and frameshift variants, as well as missense variants in conserved regions, provides a straightforward direct gene target analysis. Nevertheless, next-generation sequencing (NGS)-targeted panels for LSD-associated genes or other NGS methodologies provide a quick way to identify the molecular defect underlying diseases with such clinical variability [2]. However, in specific cases, the molecular diagnosis timeline can be even longer, when such pathogenic variants affect splicing and mRNA processing. These situations represent an additional challenge, with the identification and effect prediction of abnormal transcripts. In addition, some naturally spliced forms can raise another layer of difficulty and even mimic the molecular defect and its impact on splicing. The *IDS*, *GNPTAB*, and *NPC1* genes—whose pathogenic variants underly Mucopolysaccharidosis type II, Mucolipidosis types II or III, and Niemann–Pick type C, respectively—are some examples of LSD-associated genes with naturally occurring spliced forms already reported in the databases (https://www.uniprot.org/uniprotkb/P22304/entry#sequences (accessed on 28 September 2023)); (https://www.uniprot.org/uniprotkb/Q3T906/entry#sequences (accessed on 28 September 2023)); and (https://www.uniprot.org/uniprotkb/O15118/entry#sequences (accessed on 28 September 2023)).

Niemann–Pick type C (NPC, ORPHA: 646) in particular is a devastating neurodegenerative LSD, caused by loss-of-function variants in either the *NPC1* gene (in approximately 95% of cases) [3] or the *NPC2* gene (in 5% of cases). Analysis of next-generation sequencing (NGS) data sets indicates that the incidence rate of NPC for the classical clinical manifestations is ~1:90,000 but suggests that, for the late-onset phenotype or variant forms, the frequency might be higher [4].

Overall, the wide range of clinical phenotypes and the different ages of onset it may present with, together with the rarity of the disease and the fact it may be caused by mutations in two different genes, make its diagnosis a significant challenge. At a clinical level, NPC’s infantile forms present varying degrees of neurologic involvement and frequently present visceral manifestations, such as splenomegaly, hepatomegaly, neonatal jaundice, and hyperbilirubinemia [5,6]. Adolescent- or adult-onset NPC, on the other hand, presents with varying combinations of progressive neurologic deficits, e.g., ataxia, dystonia and/or dementia, vertical supranuclear gaze palsy (VSGP), or major psychiatric illness, including schizophrenia, depression, and psychosis, among others [6].

That is why a definitive NPC diagnosis must rely on additional laboratorial analyses. The classical method of establishing a NPC diagnosis relies on the filipin staining of cultured fibroblasts from skin biopsies [7]. This is a microscopy-based test that takes advantage of the fact that filipin specifically binds to unesterified cholesterol, allowing the evaluation of cholesterol accumulation in the perinuclear vesicular compartments [8]. This rationale is consistent with the current assumption that the impaired egress of cholesterol from the late endosome/lysosome (LE/L) is a key element of NPC pathogenesis. Nevertheless, even the most severely affected patients may fail to be diagnosed through this method [9,10]. In fact, patients with proven NPC disease may present with variable filipin patterns, from typical “classical” or “intermediate” to “atypical” or “variant” ones, which fail to be classified as a NPC by filipin staining alone. Recent advances in the field are actively contributing to an increase in the detection of NPC patients. Among those advances is the development of rapid and reliable biomarkers, including oxysterols [11,12,13], lysosphingomyelin derivatives [14,15], and bile acids [16,17], even though none of them are specific to NPC [18]. However, *N*-palmitoyl-*O*-phosphocholineserine, (PPCS, previously known as lysosphingomyelin-509) has been shown to be elevated in the plasma and dried blood spots of NPC patients [19,20]. But, these novel biomarkers are not the sole contributors to the increased recognition of this disorder and its more expedited diagnosis. The increased availability of NGS has also contributed to the update of the overall NPC diagnostic algorithm while actively contributing to an increase in the number of positive molecular NPC diagnoses. Currently, there are a number of fully described diagnostic workflows for NPC [18], which may slightly vary between different labs depending on the tests each of them has available. However, *NPC1* and *NPC2* molecular analysis is mandatory in all of them and usually represents the ultimate step towards diagnosis [21]. Indeed, a rapid molecular diagnosis of a potential NPC patient is essential, not just for swift access to available therapies (currently miglustat is the only one approved within the European Union) but also to slow the progression of the disease and ultimately because it is the sole method of offering prenatal diagnosis to affected families [22].

## 2. A Brief Overview of the Diagnostic Workflow for Niemann-Pick Type C

### 2.1. The Picture in Black and White: Standard Workflows and Straightforward Diagnoses

**Figure 1:**
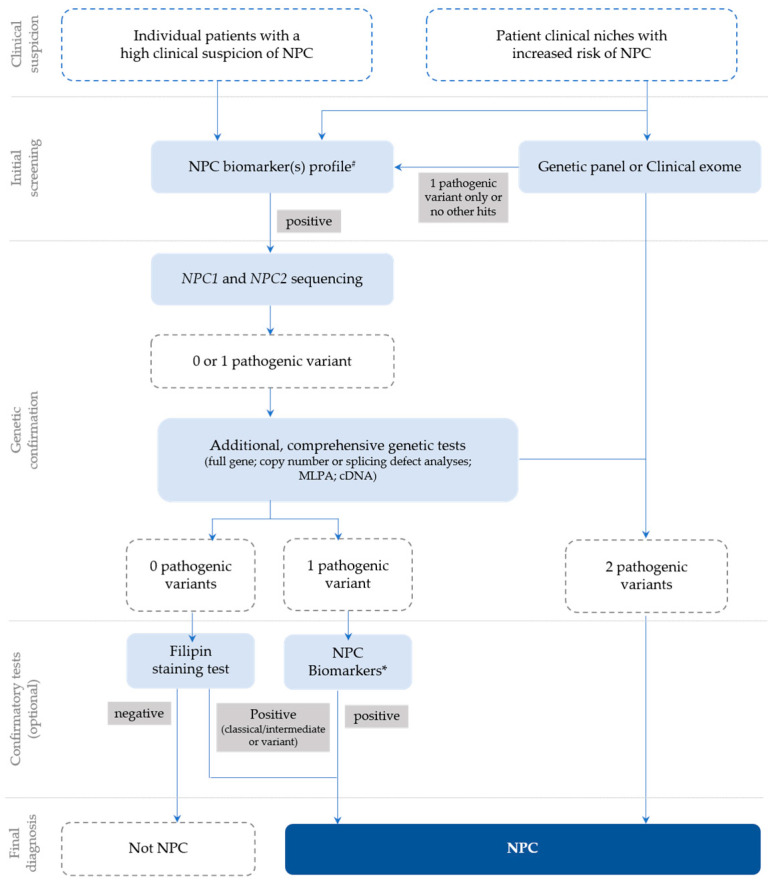
Recommendations for the detection and diagnosis of NPC, based on Patterson et al. [18] with slight updates to accommodate the most recent technologies, which are now commonly used for diagnostic purposes (e.g., clinical exome), as well as the current nomenclature. # Negative biomarkers may be suggestive that the diagnosis is not NPC; * Biomarker(s) profiling (if not initially conducted) or extended biomarker(s) profiling (in addition to those already conducted).

In general, following a suspicious timeline of clinical manifestation and/or a biomarker profile consistent with NPC, the next step is the *NPC1* and *NPC2* sequencing of the index case (Figure 1) and subsequent segregation studies of the parents [18]. The *NPC1* gene (MIM# 607623) comprises 25 exons and over 600 disease-causing variants have been reported to date [23], most of which encode missense alleles. For the *NPC2* gene (MIM# 601015), thirty-four disease-causing variants have been described and four of them are splicing (https://my.qiagendigitalinsights.com/, HGMD Professional 2023.3, accessed on 17 October 2023). Among the most common *NPC1* pathogenic variants are p.Ile1061Thr, found in 20% of patients of Western European descent [24], and p.Pro1007Ala, associated with milder forms of the disease [25,26]. In Portugal, however, the most frequent disease-causing variant is the missense p.Ala1035Val, which accounts for 15–20% of the affected cases (unpublished data transmitted by Quelhas D and Ribeiro I); it was recently reported as the most common in patients from Latin America [27].

However, the highly polymorphic nature of *NPC1* can muddle diagnostic conclusions and turn the interpretation of novel variants of unknown significance (VUSs) into a challenge. In addition, cDNA sequencing is necessary to address mRNA processing in the presence of silent variants, or other VUSs, including missense variants near the (exonic or intronic) splicing regions.

More specifically, the cDNA analysis of exonic variants may help confirm the pathogenic effect of variants predicted to affect splice sites [28]. Several splice-site pathogenic variants have been identified in NPC and in many other LSDs [29]. In some instances, these variants do not allow the generation of functional mRNAs [30]. However, they are leaky and frequently produce a small percentage of correctly spliced and translated transcripts, leading to attenuated phenotypic expression of the disease [31].

Whenever conventional gDNA analysis leads to a single variant identification, the genetic study focuses on detecting the second damaging variant. For this reason, complementary studies, such as multiplex ligation-dependent probe amplification (MLPA) in gDNA to cover intragenic deletions or duplications or cDNA sequencing, may also be required for proper diagnosis of NPC [18,22]. As straightforward as this approach may sound, reaching a conclusive molecular diagnosis of NPC may, in some cases, be harder than it seems.

### 2.2. The Grayscale Image

Among the confounding factors that can either hinder or delay a definitive diagnosis of NPC is the presence of genetic variants affecting the normal *NPC1* and *NPC2* splicing patterns.

Several pathogenic variants affecting both *NPC1* and *NPC2* mRNA splicing, occurring in intronic and exonic regions, have already been described [32]. Although quite rare, three pathogenic intronic variants have been described in the *NPC2* gene [12,28,33]. One additional variant affecting splicing was found in both healthy controls and patients [4].

Interestingly, missense variants, such as the c.1553G>A (p.Arg518Gln), were proven to have an additional impact on the splicing mechanism in the *NPC1* gene, as long as they occur in the coding exons’ splicing regulatory sequences [28,34,35,36].

**Figure 2:**
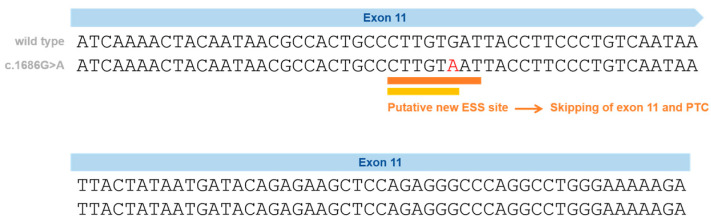
Schematic representation of the silent variants in the *NPC1* exonic region affecting splicing and the effect on splicing based on in silico predictions (Human Splicing Finder—HSF and EX-SKIP tools and Maxent). P.Val562= localization on Exon 11 (red) and the effect on splicing based on in silico predictions. EX-SKIP compares the Exonic Splicing Enhancer (ESE)/Exonic Splicing Silencer (ESS) profile of a wild type (WT) and a mutated allele to determine if a specific exonic variant increases the chance of exon skipping. It calculates the total number of ESSs, ESEs, and their ratio. The p.Val562= mutant is associated with a change in the ESE/ESS ratio, which is compatible with a higher chance of exon skipping than in the WT allele. In addition, the HSF (a tool to predict the effects of pathogenic variants on splicing signals or to identify splicing motifs in any human sequence) predicts that the p.Val562= mutant leads to the creation of an ESS site. It involves the cDNA sequences CTTGTAAT (orange) [39] and CTTGTA (yellow) [40], which might be associated with a potential alteration of splicing. In the case of the silent variant p.Val562=, functional cDNA analysis was performed [19], confirming the bioinformatic prediction of Exon 11 skippings.

Following a combined approach (gDNA and cDNA studies), we have previously proven the impact of a silent variant in the *NPC1* gene that leads to exon skipping—p.Val562= (Figure 2) [37]. This variant is located in Exon 11 and was initially reported in Spanish NPC patients and classified, at that time, as a VUS or polymorphism, after a genomic DNA study [38].

In our previous study, cDNA analysis of the affected patient and his mother (heterozygous carrier) made it possible to identify a transcript with the skipping of Exon 11. This caused a shift in the reading frame and the emergence of a premature termination codon [37], leading to its reclassification as a disease-causing variant. Importantly, the p.Val562= variant was found not only in three independent Portuguese families but also in previously reported Spanish [38] and French patients [41] (Table 1). In a French cohort, this variant was reported in heterozygosity with the p.Ile1061Thr in two siblings [41,42]; however, its functional consequence was not ascertained. Despite the sequencing of five overlapping *NPC1* cDNA fragments in the two siblings carrying the p.Val562=, the pathogenic effect of the variant was considered unknown (Supplementary Table S1, patients 25 and 25 from Nadjar et al. [41]). The most likely explanation is the degradation of the aberrant transcript by NMD. There is no information about the frequency of this variant in gnomAD. Looking to other repositories, this variant is only reported in a database from Tubingen University (NPC-db2; https://medgen.medizin.uni-tuebingen.de/NPC-db2/search.php (accessed on 28 September 2023))—it was found in one patient but not in the controls. No information was provided regarding the homo- or heterozygosity of that patient; however, both in the literature and in our cohort, only heterozygous patients were identified.

Another example of a NPC-causing variant associated with complex mRNA processing is the c.190+5G>A variant. This particular variant is located not in *NPC1*, the most obvious candidate to harbor a disease-causing mutation, but in Intron 2 of the *NPC2* gene. Again, this variant seems to be associated with a milder clinical course since both reported patients—two siblings homozygous for this variant—presented with a juvenile onset of the neurological disease and prolonged survival. A more detailed study showed that this splice variant generated multiple abnormal mRNAs [43]. However, in fibroblasts, a very small proportion of the correctly spliced transcript was also observed. Although this was not sufficient in producing enough NPC2 protein for Western Blot detection, the presence of low levels of functional protein presumably accounts for the milder clinical course. The question of whether different tissues could display variable levels of abnormally/normally spliced RNA transcribed from the c.190+5G>A variant can also be raised.

Ideally, however, these variants should be easily detected whenever an adequate cDNA analysis of any of the involved genes is performed. Still, that is not always the case, as we will demonstrate with a few practical examples.

#### 2.2.1. Splice Site Prediction Software

In light of a high NPC-suspicion index (a high suspicion score is assigned to patients who present with either two of seven key symptoms or VSGP alone) [44], the synonymous and nonsynonymous variants in either *NPC1* or *NPC2* should be studied. The predicted impact of potential splice site variants can be analyzed with the splicing prediction module of Alamut Visual software v.2.11 (Interactive Biosoftware, Rouen, France), which integrates data from three methods: Splice Site Prediction by Neural Network (NNSplice), MaxEntScan, and Human Splicing Finder (HSF) [39]. In the past few years, the SpliceAI algorithm has demonstrated the highest sensitivity and specificity when compared with other tools [45] and is nowadays the most widely recommended [46]. However, cDNA analysis is useful to study the effect of the novel variants on splicing, as well as to improve the current knowledge of the underlying molecular mechanisms and, essentially, to analyze if the variants are leaky. Other than cDNA analysis, other procedures can help to assess if a variant is leaky, namely, cloning procedures; allele-specific RNA expression quantification using PCR-based methods, among others; and procedures depending on the studied cases.

#### 2.2.2. Naturally Occurring Spliced Forms of mRNA May Mask Disease-Associated Transcripts

While analyzing cDNA samples obtained from the skin fibroblasts of NPC patients and controls, we observed the presence of an additional amplification product comprising Exons 9, 12, and 13 and missing Exons 10 and 11. This was detected both with and without cycloheximide (CHX) treatment while the pathogenic transcript due to p.Val562= was responsive to CHX. CHX is a potent NMD inhibitor that can be used to prevent the degradation of PTC-containing transcripts. In the case of disease-causing variants that affect splicing, the visualization of the aberrant transcripts can be tricky and consequently overlooked. When patients’ cells are available, the treatment of cell cultures with CHX can significantly increase the signal of the aberrant transcripts that have PTCs and are, thus, degraded by NMD but do not have any effect on the naturally occurring transcripts (a detailed protocol is described in Encarnação et al., 2020 [37].

This observation prompted us to further analyze the transcript, searching for its presence in other tissue samples. After a number of independent assessments with different primer sets and PCR conditions, it was possible to observe the presence of a smaller *NPC1* transcript, amplified in both controls and NPC patients, missing *NPC1* Exons 10 and 11. This alternative transcript may be detected after RT-PCR electrophoresis as a lower molecular weight band. This may be confusing when analyzing actual aberrant transcripts resulting from real pathogenic variants causing the constitutive splicing errors of the *NPC1* gene. This transcript lacking Exons 10 and 11 has already been reported in the Ensembl genome database (Transcript ID ENST00000591051.1).

Interestingly, this alternative skipping produces an in-frame transcript and the overall *NPC1* reading frame remains unaltered, despite missing 204 nucleotides, corresponding to 64 coding triplets.

In order to verify the expression levels of this transcript in other cells/tissues, RNA was extracted from blood samples. cDNA was then synthesized using the same amount of RNA as that used for fibroblasts samples and both transcripts were detected. Nevertheless, their expression levels in blood seemed significantly lower than those of fibroblasts. That pattern is in full agreement with previous reports on mRNA expression in normal human tissues.

**Figure 3:**
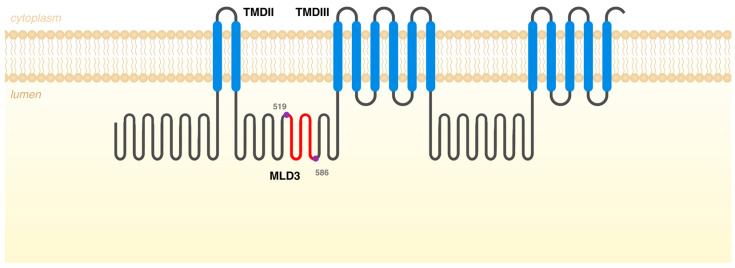
Schematic representation of NPC1 protein with the different domains. In red are the missing amino acids, if the differently spliced form would give rise to a protein. That segment is located in Middle Luminal Domain 3 (MLD3), between transmembrane domains (TMDs) II and III, and it contains the amino acids 519 to 586 (68 amino acids in total).

The presence of this naturally occurring *NPC1* alternatively spliced mRNA is actually in accordance with the in silico estimates on splice junction strengths predicted by the MaxEntScan. This tool is based on the approach for modeling the sequences of short sequence motifs, such as those involved in RNA splicing. It simultaneously scores non-adjacent as well as adjacent dependencies between positions [5]. Interestingly, when using the tool to evaluate these regions in *NPC1* Exons 9, 10, 11, and 12, we observed that the lowest 3′ss score was predicted for Exon 10 while the weakest 5′ss was predicted for Exon 11. This is in accordance with our data and can justify the existence of a naturally occurring *NPC1* transcript, which does not encompass Exons 10 and 11. If translated, one such differently spliced form would give rise to a protein, which would not include amino acids 519 to 586 (68 amino acids in total). That segment is located in Middle Luminal Domain 3 (MLD3), between transmembrane domains (TMDs) II and III of the NPC1 protein (Figure 3). MLD3 mediates the transfer of NPC2-bound cholesterol to the sterol binding domain located at the *N*-terminal domain of NPC1 and also the Ebola virus binding [47,48]. There are 20 disease-causing missense variants in this region (HGMD Professional 2023.3 in October 2023) but this is not the most conserved region of the NPC1 protein. Interestingly, however, the predicted size of that protein product would be 1210 aa but none of the transcripts listed in Ensembl match that prediction. Still, when a more detailed analysis of those transcripts is performed, namely, by Clustal omega multiple sequence alignment, it becomes evident that there is one *NPC1* transcript that does lack Exons 10 and 11 (ID ENST00000591051.1). Remarkably, however, that is not the only difference between this naturally occurring transcript and the wild type one. There is a significant difference between the transcription initiation of both transcripts, with the ENST00000591051.1 transcript Exon 1 corresponding to a sequence that partially overlaps Exon 6 of the wild-type transcript, thus comprising only 18 coding exons, in opposition to the wild-type one, which spans 25 exons.

This is yet another reason for us to highlight its presence under some amplification conditions as it may confound the analysis of cDNA patterns in that region, with serious implications for the classification of pathogenic variants predicted to impact splicing.

## 3. Variants in the *NPC1* Gene That Affect Splicing

Pathogenic variants that affect pre-mRNA splicing account for at least 15% of disease-causing mutations [49]. Most of these variants affect 5′ and 3′ss, the polypyrimidine tract, the branch-point sequence, and also *cis*-acting elements (exonic/intronic splicing enhancers and silencers). Other variants create novel splicing sequences deeply within introns, causing the abnormal inclusion of intron sequences. All of these variants lead to the production of abnormal transcripts that usually contain PTCs and are degraded by nonsense-mediated mRNA decay (NMD) [50]. Even exonic variants (missense and synonymous) may affect splicing, having a completely different effect from what was expected [51]. Therefore, both gDNA and cDNA should be analyzed.

The *NPC1* gene contains 25 exons and spans 55 kb from the base pairs (23, 531, 442) to 23, 586, 506 on the reverse strand of chromosome 18 at 18q11.2 (NC_000018.10); here we report on 53 published pathogenic variants affecting splicing.

Seven of them are exonic, two are synonymous, five are missense (Table 1 and Table 2), and forty-six are intronic variants (Table 3), mainly affecting the 3′ss and the 5′ss; however, there are also variants reported in the branch point as well as deep-intronic variants.

Altogether, these observations call attention to the need for extensive mRNA studies in *NPC1*, or even *NPC2*, to establish a definitive NPC diagnosis. In this context, the presence of an alternatively spliced transcript may be somewhat confusing and even mask or mimic a real pathogenic variant that impacts splicing only in NPC patients and *NPC1* variant carriers.

As for tissue-specific differences in the relative abundance of the two *NPC1* splice isoforms, we observed a higher expression of the spliced isoform in fibroblasts than in blood. In fact, in the genotype-tissue expression project (GTEx), which studies tissue-specific gene expression and regulation in fibroblasts, they quantified 30 fragments per kilobase of exon per million fragments mapped; meanwhile, in whole blood, only 17 fragments were quantified (https://www.genecards.org/cgi-bin/carddisp.pl?gene=NPC1&keywords=npc1#expression (accessed on 28 September 2023)).

## 4. Conclusions

Altogether, our data highlight the fact that a naturally occurring spliced form of *NPC1* mRNA should be taken into consideration when analyzing the *NPC1* cDNA amplicons, especially when considering fibroblast cell cultures. Even though the traditional diagnostic workflows rely almost exclusively on targeted genomic DNA sequencing, the study of mRNA processing is often critical to understanding the real impact of the genomic variant. Therefore, cDNA analysis is highly recommended as the presence of an alternative transcript can muddle the results. Thus, one should be aware that, under certain conditions relying on the amount of total RNA used for in vitro cDNA synthesis, the transcript here reported is naturally occurring and not related to disease.

For now, the physiological role of such a transcript can only be a speculative assumption. However, the biological role of the NPC1 protein is still not fully understood. Specific efforts are striving to better understand how this protein, and others (including NPC2), take part in the egress of unesterified cholesterol from the LE/L compartment. Moreover, other important roles for NPC1, such as being the receptor of the Ebola virus, have recently been uncovered. Therefore, this transcript, as well as the encoding, may have a biological role that merits deeper research far beyond this technical recommendation for the diagnosis of NPC disease.

## Figures and Tables

**Table 1:** Patients identified as compound heterozygous for the splicing variant c.1686G>A in the *NPC1* gene.

Patient	Origin	Genotype	Age at Neurologic Onset (Years)	Clinical Form	Filipin Staining	PPCS# (Control Range 0.3–3.9 MoM)	Reference
P1	Portugal	c.1514T>G (p.Val505Gly) c.1686G>A (p.Val562=)	6	Juvenile	Variant	12.4	[37]
P2	Portugal	c.1552C>T (p.Val518W) c.1686G>A (p.Val562=)	7	Juvenile	Variant	4.3	[37]
P2′	Portugal	c.1552C>T (p.Val518W) c.1686G>A (p.Val562=)	7	Juvenile	Variant	3.2	[37]
P3	Portugal	c.1686G>A (p.Val562=) c.3019C>G (p.Pro1007Ala)	18	Adult	n.a.	6.4	This study
P4	Spain	c.1436G>A (p.Cys479Tyr) c.1686G>A (p.Val562=)	9	Juvenile	Variant	n.a.	[38]
P5	France	c.1686G>A (p.Val562=) c.3182T>C (p.Ile1061Thr)	18	Adult	Intermediate	n.a.	[41]
P5′	France	c.1686G>A (p.Val562=) c.3182T>C (p.Ile1061Thr)	18	Adult	Intermediate	n.a.	[41]

Patients P2 and P2′, P5 and P5′ are siblings, GeneBank references: *NPC1*: NM_000271.5, Transcript ENST00000269228.10, n.a.: information not available/not applicable, MoM: multiple of median, # *N*-palmitoyl-*O*-phosphocholineserine (PPCS): formerly known as lyso-SM-509, n.a.: information not available/not applicable.

**Table 2:** Exonic splicing variants in the *NPC1* gene.

Splicing Variant	Location	Impact on mRNA and Protein Levels	Clinical Form	Reference
c.1553G>A	Exon 9	Changes amino acid and induces a splicing error. The variant occurs in the last nucleotide of Exon 9. It induces the skipping of Exon 9 (227 bp-deletion), leading to a 75 amino acid deletion and a frameshift, creating a premature termination codon (PTC) at position 499 of Exon 10.	Late infantile	[36]
c.2292G>A (also known as IVS16-82G>A)	Exon 15	Silent variant (p.Ala764=). Creation of a splice site in Exon 15, generating a transcript with a 48 bp in-frame deletion (750del16aa).	n.a.	[26]
c.2599C>T	Exon *17*	Changes amino acid but also induces a splicing error. In silico prediction, only by Neural Network Splice Site Prediction.	n.a.	[28]
c.2911G>C	Exon 19	Changes amino acid but also induces a splicing error. In silico prediction, only by Neural Network Splice Site Prediction.	n.a.	[28]
c.3422T>G	Exon 22	Activation of a new donor splice site in which the 3′ end of Exon 22 is deleted.	n.a.	[34]
c.3754G>C (previously described as p.Gly1252Arg)	Exon 24	Affects the last nucleotide of Exon 24 generating skipping of Exon 24.	Late infantile	[38,52]

n.a.: information not available/not applicable.

**Table 3:** Intronic splicing variants in *NPC1* gene.

Genotype	Location	Impact on mRNA and Protein Levels (as Described in the Reference Paper)	Age at Neurologic Onset (Years)/Clinical Manifestation	Ref
c.57+4A>G	Intron 1	No cDNA analysis was performed. Only in silico analysis was described.	Ataxia at the age of 20.	[53]
c.58−3T>G	Intron 1	No cDNA analysis was published.	Identified in compound heterozygosity with p.Val1165Met in two siblings from an Italian cohort. One presents a late infantile form and the other a juvenile form.	[54]
c.58-3281C>G	Intron 1	Pseudoexon insertion, creating a novel acceptor region and activating a cryptic donor splice site.	n.a.	[52]
c.58-3290G>A	Intron 1	Partial retention of Intron 1.	At 12 years old when in compound heterozygosity with the p.Gly992Arg variant.	[41]
c.181-6T>A	Intron 2	Skipping of Exon 2.	At 20 years old when in compound heterozygosity with the p.Tyr475Cys variant.	[41]
c.181-2A>G	Intron 2	Generation of a novel splicing acceptor site located one nucleotide (NT) upstream of the canonical 3′ss. Thus, the last NT of Intron 2 is retained within the mature transcript, causing a frameshift in the open reading frame and the generation of a PTC that would eventually result in the synthesis of a truncated protein.	At 40 years when in compound heterozygosity with the variant Gly1012Cys.	[41,54]
c.287+1G>A	Intron 3	Located at the 5′ donor splice site of Intron 3. This location has a 0.98 score prediction as a splice site (Neural Network) and a confidence of 0.76 as a donor splice site (NetGene2), likely disturbing normal splicing and altering protein features (Mutation Taster). According to recommendations of the ACMG, this variant was classified as pathogenic.	At 4 months when in compound heterozygosity with the variant (p.Arg1186His). NPC was confirmed by a filipin test on a fibroblast cell culture at 4.5 years old.	[55]
c.464-2A>C	Intron 4	Affects the 3′ acceptor splice site of Intron 4, leading to the activation of the downstream cryptic splice site (score, 0.98), predicted to be a genomic region overlapping Exon–Intron 5. A possible consequence could be an unstable aberrantly spliced transcript, carrying a premature translation stop codon, possibly subjected to NMD. cDNA analysis showed degradation of the patient’s mRNA.	Identified in two siblings. The second disease-causing variant was not identified. First neurologic symptoms at 2 and 3 years old.	[56]
c.882-40T>A	Intron 6	n.a.	At 12 years, identified in compound heterozygosity with the p.Ile1061Thr variant.	[57]
c.882-28A>G	Intron 6	It occurs in a conserved adenosine of a putative branch point sequence. The Exon 6–Exon 8 junction in this mRNA causes a frameshift and a premature stop codon, predicted to result in a truncated protein. The skipping of Exon 7 was confirmed both in the patient´s fibroblasts (cDNA analysis) and in cells expressing minigenes.	Identified in two siblings in compound heterozygosity with p.Arg978Cys, both with the first neurological symptoms occurring during their 20 s.	[58]
c.882-28A>T	Intron 6	The conserved adenosine residue of the lariat branch point in Intron 6 causes an abnormally spliced mRNA with the complete skipping of Exon 7 (c.882_954del73). The loss of Exon 7 disrupts the reading frame, leading to a PTC, which activates mRNA degradation by the NMD process. This was confirmed by cDNA analysis.	Identified in one Spanish patient with the late infantile form in compound heterozygosity with p.Ser425X.	[52]
c.881+3A>G	Intron 6	No cDNA analysis was performed but bioinformatics analysis predicts that the variant affects the splicing donor.	Identified in two patients (two independent studies), both presenting development regression of movement and intelligence.	[59]
c.955+1G>A	Intron 7	No cDNA analysis was performed.	Identified in compound heterozygosity with p.Trp942Cys in one patient with severe infantile disease (symptoms at 1 year old).	[38]
c.955+5G>A	Intron 7	No cDNA analysis was performed.	Identified in compound heterozygosity with p.Pro1007Ala in a patient with their first neurological symptoms at 1 year old.	[60]
c.1554-1009G>A	Intron 9	Creates a cryptic donor splice site, resulting in the incorporation of 194 bp of Intron 9 as a new exon (pseudoexon) in the mRNA. This new transcript bears a premature termination codon and is degraded by the NMD mechanism. This was observed in the patient’s fibroblasts and also in HeLa cells transfected with a mutant but not with a wild-type *NPC1* minigene.	Compound heterozygous with the in-frame deletion insertion p.Asn961_Phe966delinsSer.	[61]
c.1553+1G>C	Intron 9	n.a.	Identified in compound heterozygosity with p.Ser652Trp.	[62]
c.1553+1G>A	Intron 9	n.a.	Identified in one patient with the early infantile form and in compound heterozygosity with c.181-2A>G (p.Glu61GlyfsTer24).	[54]
c.1553+5G>A	Intron 9	cDNA analysis showed the skipping of Exon 9.	Identified in three Turkish cases from the same family.	[63]
c.1654+1G>T	Intron 10	n.a.	Infantile.	[3]
c.1757+2T>C	Intron 11	n.a.	n.a.	[64]
c.1757+5G>A	Intron 11	n.a.	Identified in a Chinese patient.	[65]
c.1947+2T>G	Intron 12	*In silico* by Neural Network Splice Site Prediction.	n.a.	[28]
c.1947+5G>C	Intron 12	Predicted disruption of the Intron 12 donor site and subsequent use of a cryptic donor site within Exon 12, resulting in an alternative transcript. As a consequence, there is a 25 amino acid in-frame deletion (p.Ile626_Val650del) that, based on in silico modeling, may disrupt the third transmembrane domain of the NPC1 protein. This was validated in *NPC1* cDNA.	Identified in a NPC1 cell line fibroblast maintained by the Coriell Repository (GM03123).	[66]
c.2130+1G>A	Intron 13	n.a.	Found in homozygosity in three patients from Saudi Arabia.	[67]
c.2130+2T>C	Intron 13	No cDNA analysis was conducted.	Found in two siblings in compound heterozygosity with the p.Pro1007Ala variant.	[12]
c.2246-2A>G	Intron 14	*In silico* by Neural NetworkSplice Site Prediction.	n.a.	[28]
c.2245+1G>A	Intron 14	n.a.	Found in compound heterozygosity with p.Pro543Leu in a patient with the early infantile form.	[68]
c.2374-1G>A	Intron 15	n.a.	Found in a Whole Genome Sequencing project of a large cohort.	[69]
c.2604+1G>A	Intron 17	n.a.	Found in an Indian patient in compound heterozygosity with p.Ile690Phe.	[70]
c.2604+2T>G	Intron 17	n.a.	Found in one Indian patient in homozygosity. The cohort included patients with predominant central nervous system white matter abnormalities.	[71]
c.2604+5G>A	Intron 17	Located in a conserved position of the donor splice site of Intron 17. Promotes the skipping of Exon 17.	Neonatal (when present in both alleles).	[52]
c.2795+1G>C	Intron 18	Located in the 5′ donor splice site of Intron 18. qRT-PCR analysis of the *NPC1* mRNA from the patient´s fibroblasts showed that this splicing variant generates an unstable mRNA that is most likely degraded by NMD.	Identified in compound heterozygosity with the p.Val1165Met variant.	[72]
c.2795+5G>A	Intron 18	No cDNA analysis was conducted.	Identified in compound heterozygosity with p.Leu107CysfsTer5. Diagnosed at 1 year old.	[73]
c.2795+5G>C	Intron 18	It decreases the strength of the canonical donor splicing site and the strongest donor splice site is predicted to be 124 bp downstream. The analysis of cDNA sequence encompassing Exons 17–20 from a patient carrying this variant confirmed the inserted fragment of 124 bp (between Exons 18 and 19).	Identified in a Serbian patient in compound heterozygosity with the splicing c.2819C>T.	[74]
c.2912-3C>G	Intron 19	Predicted to alter the splicing acceptor site of Intron 19 (*in silico* analysis).	Identified in a Chinese patient with the late infantile form in compound heterozygosity with the c.2302_2303insG.	[75]
c.3041+5G>A	Intron 20	n.a.	n.a.	[12]
c.3246-2A>G	Intron 21	n.a.	Identified in compound heterozygosity with p.Arg978Cys in two siblings with NPC.	[76]
c.3477+1G>A	Intron 22	n.a.	Identified in a French patient in compound heterozygosity with p.Ala1174Val.	[3]
c.3477+4A>G	Intron 22	n.a.	Identified in compound heterozygosity with p.Pro1007Ala. The age at onset was 29 years old, with ataxic gait as the first symptom. Also presenting cerebellar ataxia, dysarthria/dysphagia, VSGP, pyramidal tract signs, executive dysfunction, and the magnetic resonance imaging showing cerebellar atrophy.	[77]
c.3591+1G>A	Intron 23	Activation of a splice site in Intron 23, which is predicted to lead to a 45 bp in-frame insertion and the activation of a splice site in Exon 23, leading to a 63 bp in-frame deletion. In cells from patients homozygous for the variant, no detectable band was observed in the NPC1 protein region, showing that none of the two predicted transcripts produces protein.	Identified in homozygosity in a Portuguese patient with the early infantile form (age at neurological onset was 2 years). Severe hepatosplenomegaly. Sibling deceased at 3 months from the severe neonatal rapidly fatal cholestatic form alongside pulmonary infiltration.	[26]
c.3591+1G>T		n.a.	n.a.	[78]
c.3591+3G>C	Intron 23	n.a.	Identified in compound heterozygosity with p.Asp944Asn.	[62]
c.3591+5G>A	Intron 23	Three abnormally spliced cDNAs were observed/identified: one with an insertion of the first 45 bp of Intron 23 (c.3591_3592ins45), another with a deletion of the last 63 bp of Exon 23 (c.3529_3591del63), and a third one with the skipping of Exon 23 (c.3478_3591del114).	Identified in homozygosity in a Spanish patient with a neonatal form.	[52]
c.3754+1G>A	Intron 24	n.a.	n.a.	[12]
c.3754+1G>C	Intron 24	Skipping of Exon 24, frameshift, and PTC.	Identified in heterozygosity with p.Leu724Pro. Late infantile clinical form.	[25]
c.3754+3A>C	Intron 24	n.a	n.a	[12]

n.a.: information not available/not applicable, PTC: premature termination codon.

## Data Availability

The data presented in this study are available on request from the corresponding author.

## Abbreviations

ACMG	American College of Medical Genetics and Genomics
CHX	Cycloheximide
ESE	Exonic splicing enhancer
ESS	Exonic splicing silencer
GTEx	Genotype-tissue expression project
HSF	Human Splicing Finder
LE/L	Late endosome/lysosome
LSD	Lysosome storage disorder
MLPA	Multiplex ligation-dependent probe amplification
MoM	Multiple of median
MLD3	Middle luminal domain 3
NGS	Next-generation sequencing
NPC	Niemann-Pick type C
NMD	Nonsense-mediated mRNA decay
PPCS	N-palmitoyl-O-phosphocholineserine
PTC	Premature termination codon
TMD	Transmembrane domain
VUS	Variant of unknown significance
VSGP	Vertical supranuclear gaze palsy
wt	Wild type
